# Rapid micellar HPLC analysis of loratadine and its major metabolite desloratadine in nano-concentration range using monolithic column and fluorometric detection: application to pharmaceuticals and biological fluids

**DOI:** 10.1186/s13065-016-0225-5

**Published:** 2016-12-01

**Authors:** Fathalla Belal, Sawsan Abd El-Razeq, Mohamed El-Awady, Sahar Zayed, Sona Barghash

**Affiliations:** 1 Pharmaceutical Analytical Chemistry Department, Faculty of Pharmacy, Mansoura University, Mansoura, 35516 Egypt; 2 Analytical Chemistry Department, Faculty of Pharmacy (Girls),, Al-Azhar University, Cairo, 11754 Egypt; 3 Unit of Drug Analysis, Faculty of Pharmacy, Mansoura University, Mansoura, 35516 Egypt

**Keywords:** Loratadine, Desloratadine, Micellar monolithic HPLC, Fluorometric detection, Tablets, Biological fluids

## Abstract

**Background:**

Loratadine is a commonly used selective non-sedating antihistaminic drug. Desloratadine is the active metabolite of loratadine and, in addition, a potential impurity in loratadine bulk powder stated by the United States Pharmacopeia as a related substance of loratadine. Published methods for the determination of both analytes suffer from limited throughput due to the time-consuming steps and tedious extraction procedures needed for the analysis of biological samples. Therefore, there is a strong demand to develop a simple rapid and sensitive analytical method that can detect and quantitate both analytes in pharmaceutical preparations and biological fluids without prior sample extraction steps.

**Results:**

A highly-sensitive and time-saving micellar liquid chromatographic method is developed for the simultaneous determination of loratadine and desloratadine. The proposed method is the first analytical method for the determination of this mixture using a monolithic column with a mobile phase composed of 0.15 M sodium dodecyl sulfate, 10% *n*-Butanol and 0.3% triethylamine in 0.02 M phosphoric acid, adjusted to pH 3.5 and pumped at a flow rate of 1.2 mL/min. The eluted analytes are monitored with fluorescence detection at 440 nm after excitation at 280 nm. The developed method is linear over the concentration range of 20.0–200.0 ng/mL for both analytes. The method detection limits are 15.0 and 13.0 ng/mL and the limits of quantification are 20.0 and 18.0 ng/mL for loratadine and desloratadine, respectively. Validation of the developed method reveals an accuracy of higher than 97% and intra- and inter-day precisions with relative standard deviations not exceeding 2%.

**Conclusions:**

The method can be successfully applied to the determination of both analytes in various matrices including pharmaceutical preparations, human urine, plasma and breast milk samples with a run-time of less than 5 min and without prior extraction procedures. The method is ideally suited for use in quality control laboratories. Moreover, it could be a simple time-saving alternative to the official pharmacopeial method for testing desloratadine as a potential impurity in loratadine bulk powder.

**Graphical abstract:**
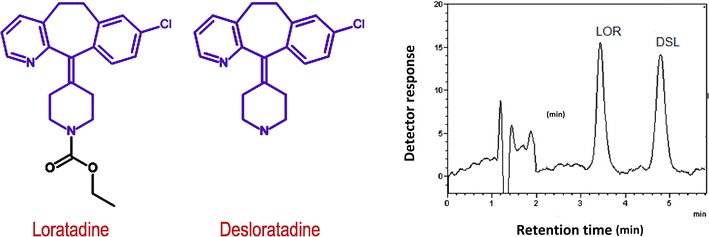
Typical chromatogram of loratadine and its major metabolite desloratadine using the proposed micellar HPLC method

## Background

Allergies are one of the four most common issues for public health along with tumors, cardiovascular diseases and AIDS. Each decade, a dramatic rise in allergies is observed in most countries. Histamine H1-receptor antagonists are the foremost known therapeutic agents used in the control of allergic disorders [1].

**Fig. 1:**
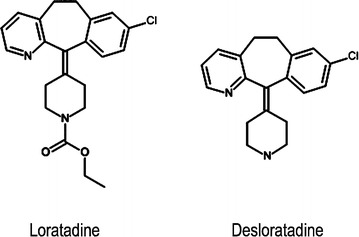
Chemical structures of the studied analytes

Loratadine (LOR) (Fig. 1) is a commonly used selective non-sedating H1-receptor antagonist which is not associated with performance impairment [2]. Desloratadine (DSL) (Fig. 1), the descarboethoxy form and the major active metabolite of LOR, is also a non-sedating H1-receptor antagonist with an antihistaminic activity of 2.5–4 times as great as LOR [3]. Moreover, DSL is a potential impurity in LOR bulk powder stated by the United States Pharmacopeia [4] as a related substance of LOR. Chemically, both LOR and DSL are weak bases. The pKa of LOR is 5.25 at 25 °C while DSL has two pKa’s, 4.41 and 9.97 at 25 °C [5]. The octanol/water partition coefficient log *P* of LOR is 5 [6] while of DSL is 3.2 [7]. The high similarities between LOR and DSL regarding structure and physicochemical properties renders their simultaneous analysis challenging. Different analytical methods have been published for the simultaneous determination of LOR and DSL including UPLC [8], HPLC [9–24], HPTLC [25], TLC [26], GC [27] spectrophotometric [28] and capillary electrophoretic [29] methods. The main drawback of these methods is the limited throughput due to required time-consuming steps. Considering biological applications, the reported methods for the analysis of LOR and DSL in biological fluids involve tedious and time-consuming preparative steps such as protein precipitation, liquid–liquid or solid-phase extraction and evaporation prior to the chromatographic separation. Therefore, there is still a strong demand to develop a simple rapid and sensitive analytical method that can detect and quantitate both analytes in pharmaceutical preparations and biological fluids without the need for sample pretreatment procedures.

The use of chromatographic methods for pharmaceutical analysis in comparison to other analytical methods has several advantages including high versatility, selectivity and efficiency, in addition to its ability to be coupled with different sample extraction techniques [30–33]. Micellar liquid chromatography (MLC) is advantageous over conventional liquid chromatography due to several reasons including the smaller concentration of organic solvent in the mobile phase which render it cheaper and less toxic, the improved selectivity and ability to separate different hydrophobic and hydrophilic analytes due to variable mechanisms of interaction between analytes and the mobile and stationary phases, the excellent solubilizing power of micelles and the ability to use direct injection of complex sample matrices including biological fluids without pretreatment procedures [34–36]. Monolithic silica is one of the new types of sorbents used in liquid chromatography. It is characterized by the ability to separate complicated sample mixtures with a very high efficiency and very short retention times using high flow rates with minimal back pressure due to the high porosity and permeability of the monolith as well as the presence of small-sized skeletons [37, 38].

The current study describes a novel, simple, sensitive and environment-friendly MLC–monolithic method for the simultaneous determination of LOR and DSL in Tablets and in spiked human plasma, urine and breast milk using fluorescence detection with a run-time of less than 5 min. To the best of our knowledge, the proposed method is the first MLC-monolithic method for the analysis of this mixture.

## Experimental

### Apparatus

Chromatographic measurements were performed with a Shimadzu LC-20AD Prominence liquid chromatograph (Japan) equipped with a Rheodyne injection valve (20-µL loop) and a RF-10AXL fluorescence detector. A Consort NV P-901 pH meter (Belgium) was used for pH measurements.

### Materials and reagents

All the chemicals used were of Analytical Reagent grade, and the solvents were of HPLC grade. Loratadine (certified purity 99.7%) and desloratadine (certified purity 99.6%) were kindly provided by Schering-Plough Co., USA. Sodium dodecyl sulfate (SDS) was obtained from Merck KGaA (Darmstadt, Germany). Triethylamine (TEA) and orthophosphoric acid, 85% were obtained from Riedel-de Haën (Seelze, Germany). Methanol, ethanol, n-propanol, *n*-Butanol and acetonitrile (HPLC grade) were obtained from Sigma-Aldrich (Germany).

Pharmaceutical preparations containing the studied drugs were purchased from the local Egyptian market. These include Loratadine 10 mg Tablets labeled to contain 10 mg of LOR (produced by Misr Company for Pharmaceutical Industries, Cairo, Egypt, batch#150103), Desa 5 mg Tablets labeled to contain 5 mg of DSL (produced by Delta Pharma Tenth of Ramadan City, Egypt, batch#31910).

The human plasma sample was kindly provided by Mansoura University Hospitals, Mansoura, Egypt and kept frozen at −5 °C until use after gentle thawing. Drug free urine sample was collected from a male healthy adult volunteer (30-years old). The breast milk sample was obtained from a female healthy volunteer (28-years old).

### Chromatographic conditions

A Chromolith^®^ Speed RODRP-18 (Merck, Germany) end-capped column (100 mm × 4.6 mm) was used in this study. The micellar mobile phase consisted of 0.15 M sodium dodecyl sulfate, 0.3% TEA and 10% *n*-Butanol in 0.02 M orthophosphoric acid, adjusted at pH 3.5. The mobile phase was filtered through 0.45-µm Millipore membrane filter and degassed by sonication for 30 min before use. The separation was performed at room temperature with a flow rate of 1.2 mL/min and fluorescence detection at 440 nm after excitation at 280 nm.

### Standard solutions

Stock solutions containing 200.0 μg/mL of each of LOR and DSL in methanol were prepared and used for maximum one week when stored in the refrigerator. Working standard solutions were prepared by appropriate dilution of the stock solutions with the mobile phase.

### General procedure and construction of the calibration graphs

Accurately measured aliquots of the stock solutions were transferred into a series of 10-mL volumetric flasks and completed to volume with the mobile phase so that the final concentrations of the working standard solutions were in the range of 20–200 ng/mL for both LOR and DSL. The standard solutions were then analyzed by injecting 20 μL aliquots (triplicate) and separation under the optimum chromatographic conditions. The average peak area versus the final concentration of the drug in ng/mL was plotted to get the calibration graphs and then linear regression analysis of the obtained data was performed.

### Analysis of pharmaceutical preparations

An accurately weighed amount of the mixed contents of 20 finely powdered tablets equivalent to 10.0 mg of LOR or 5.0 mg of DSL was transferred into a 50.0-mL volumetric flask and about 20 mL of methanol was added. The flasks were then sonicated for 30 min, completed to the mark with methanol and filtered through a 0.45-μm membrane filter. Further dilution with the mobile phase was done to obtain the working standard solution to be analyzed as described under the section “General procedure and construction of calibration graphs”. The recovered concentration of each analyte was calculated from the corresponding regression equation.

### Analysis of spiked biological fluids

New calibration graphs were constructed using spiked biological fluids as follows: 1 mL aliquots of human urine, plasma or breast milk samples were transferred into a series of 10-mL volumetric flasks, spiked with increasing concentrations of LOR and DSL and then completed to the mark with the mobile phase and mixed well (final concentration was in the range of 5.0–50.0 ng/mL for both analytes). The solution were then filtered through a 0.45-μm membrane filter and directly injected into the chromatographic system under the above described chromatographic conditions. The linear regression equations relating the peak areas to the concentration (ng/mL) were derived for each analyte.

## Results and discussion

**Fig. 2:**
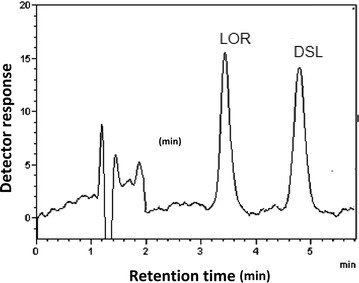
Typical chromatograms of a synthetic mixture of LOR and DSL (25 ng/mL of each) under the described chromatographic conditions: 0.15 M sodium dodecyl sulphate, 0.3% TEA, 10% 1-butanol in 0.02 M orthophosphoric acid, pH 3.5 and a flow rate of 1.2 mL/min

The proposed MLC method allows the simultaneous determination of LOR and DSL in pure form, tablets and biological fluids. Figure 2 illustrates a typical chromatogram for the analysis of a prepared mixture of LOR and DSL under the above described optimum chromatographic conditions, where well-separated symmetrical peaks were observed. The migration order of analytes can be interpreted in terms of the electrostatic interaction between analytes and the SDS monomers adsorbed on the stationary phase. In MLC, the main changes in the observed chromatographic performance are due to the adsorption of surfactant monomers on the stationary phase [36]. The modified stationary phase with SDS monomers is negatively charged and the studied analytes are positively charged at the mobile phase pH (3.5) which indicates a strong electrostatic attraction to the stationary phase. According to the pKa values of the analytes, DSL is doubly protonated at the mobile phase pH while LOR has a single positive charge. Therefore, the interaction of DSL with the stationary phase is stronger and so its retention time is longer.

As starting chromatographic conditions, the following mobile phase was utilized: 0.15 M sodium dodecyl sulfate, 0.3% TEA and 10% n-propanol in 0.02 M orthophosphoric acid, adjusted to pH 6.0 with a flow rate of 1.0 mL/min and using 290 nm as an excitation wavelength and 438 nm as an emission wavelength. Optimization of the experimental parameters affecting the selectivity and efficiency of the MLC system was performed by changing each in turn while keeping other parameters constant as shown in the following sections:

### Method development

#### Choice of column

Two different columns were tested including: Chromolith^®^ Speed ROD RP-18 (Merck, Germany) end-capped column (100 mm × 4.6 mm) and Chromolith^®^ Speed ROD RP-18 (Merck, Germany) end-capped column (50 mm × 4.6 mm). The first column showed better results where the peaks of both analytes were more symmetrical and well-defined with a total run time less than 5 min.

#### Choice of detection wavelength

The fluorescence behavior of both LOR and DSL was carefully studied in order to define the optimum wavelength combination. The best sensitivity was achieved when 280 nm was used as the excitation wavelength and 440 nm as the emission wavelength.

#### Effect of mobile phase composition

For optimum chromatographic separation, the effect of variation of the mobile phase composition was intensively studied in order to achieve the highest selectivity and sensitivity of the developed method within a short analysis time. The study included the effect of variation of pH, variation of surfactant concentration and variation of type and concentration of the organic modifier. A summary of the results of this optimization study is presented in Table 1.

**Table 1:** Optimization of the chromatographic conditions for separation of the studied analytes by the proposed HPLC method

Parameter	No. of theoretical plates		Resolution	Tailing factor		Selectivity factor (α)
	LOR	DSL		LOR	DSL	
pH of the mobile phase						
2.5	1707	1738	3.25	1.34	1.23	1.75
3.0	1811	1910	3.41	1.32	1.22	1.90
3.5	2328	2693	4.93	1.31	1.21	1.94
4.0	1601	2433	2.13	1.39	1.29	1.32
5.0	1908	2508	2.94	1.44	1.3	1.63
6.0	Unresolved peaks					
SDS concentration (M)						
0.05	1584	1667	5.66	1.58	1.47	2.26
0.1	1453	1627	4.91	1.52	1.44	1.94
0.12	1501	2133	4.72	1.3	1.23	1.87
0.15	2328	2693	4.93	1.31	1.21	1.94
0.175	1918	2216	3.64	1.45	1.28	1.42
*n*-Butanol concentration (%v/v)						
5%	692	727	4.52	2.03	1.77	1.76
8%	1575	1825	4.12	1.29	1.28	1.71
10%	2328	2963	4.93	1.31	1.21	1.94
12%	1487	1938	3.83	1.45	1.33	1.59
Flow rate (mL/min)						
0.8	2190	2105	5.36	1.36	1.34	2.21
1.0	1887	2328	5.12	1.39	1.29	2.16
1.2	2328	2693	4.93	1.31	1.21	1.94
1.5	1558	2117	3.93	1.20	1.14	1.62
Column temp.						
25	2328	2693	4.93	1.31	1.21	1.94
35	1962	2206	4.02	1.98	2.11	1.77
45	884	679	5.48	2.23	2.4	2.20

##### Variation of pH of the mobile phase

The pH of the mobile phase was changed over the range of 2.5–6.0. As shown in Table 1, pH 3.5 was found to be the optimum pH showing well-resolved symmetrical peaks with the highest number of theoretical plates and highest resolution within a short run time.

##### Variation of surfactant concentration

The influence of different concentrations of SDS (0.05–0.175 M) on the selectivity, resolution and retention times of the studied analytes was investigated. By increasing the SDS concentration, the retention times of both analytes were decreased with better peak symmetry. As presented in Table 1, 0.15 M SDS was found to be the optimum giving the highest number of theoretical plates and the highest resolution.

##### Variation of type and concentration of the organic modifier

Different organic modifiers were investigated including acetonitrile, methanol, ethanol, n-propanol and *n*-Butanol. The best organic modifier was found to be *n*-Butanol showing satisfactory resolution and efficiency within a short run time (less than 5 min). The use of acetonitrile, methanol, ethanol or n-propanol resulted in an increase in the retention time for both analytes with a decrease in the number of theoretical plates compared to the use of *n*-Butanol. That is because the addition of these solvents increases the polarity of the mobile phase relative to *n*-Butanol and since the studied analytes are hydrophobic compounds; this lead to an increase in the retention time for both analytes which is associated with larger peak width and lower number of theoretical plates.

The effect of variation of *n*-Butanol concentration on the chromatographic behavior of the studied analytes was investigated in the concentration range of 5.0–12.0%. Based on the results obtained (see Table 1), 10.0% *n*-Butanol was found to be the optimum concentration regarding separation efficiency and resolution.

#### Effect of flow rate

Table 1 shows the effect of different flow rates (0.8–1.5 mL/min) the chromatographic separation. A flow rate of 1.2 mL/min was chosen to be the optimum as it shows the highest efficiency in a short analysis time. Although lower flow rates showed higher resolution they were not selected as they lead to an increase in the total run time in addition to a decrease in the number of theoretical plates for both analytes.

Based on the above measurement series, the optimum chromatographic conditions were as follows:

The micellar mobile phase consists of 0.15 M sodium dodecyl sulfate, 0.3% TEA and 10% *n*-Butanol in 0.02 M orthophosphoric acid, adjusted at pH 3.5. A monolithic C18 column was utilized. The separation was performed at room temperature with a flow rate of 1.2 mL/min and fluorescence detection at 440 nm after excitation at 280 nm.

### Validation of the method

Validation of the developed HPLC method was performed according to the international conference on harmonization (ICH) Guidelines [39]. Different validation characteristics were investigated as follows:

#### Linearity

The linearity of the developed method was confirmed by plotting the peak area against the analyte concentration in ng/mL. The graphs were linear over the concentration range of 20.0–200.0 ng/mL for both analytes. Linear regression analysis of the obtained data gave the following regression equations:

P = −24.518 + 1.844C (*r* = 0.9999) for LOR, P = −18.97 + 1.749C (*r* = 0.9999) for DSL.

Where P is the peak area, C is the analyte concentration in ng/mL and *r* is the correlation coefficient. Statistical analysis [40] of data showed high values of *r*, small values of the standard deviation of residuals (S_y/x_), of intercept (S_a_) and of slope (S_b_), and small values of the percentage relative standard deviation which indicate linearity of the developed method over the studied concentration range (Table 2).

**Table 2:** Analytical performance data for the determination of the studied analytes by the proposed method

Parameter	LOR	DSL
Linearity range (ng/mL)	20.0–200.0	20.0–200.0
Intercept	−24.518	−18.97
Slope	1.844	1.749
Correlation coefficient (*r*)	0.9999	0.9999
SD of residuals (S_y/x_)	1.140	0.856
SD of intercept (S_a_)	0.9054	0.679
SD of slope (S_b_)	8.314 × 10^−3^	6.243 × 10^−3^
% RSD^a^	1.08	1.17
MDL (ng/mL)^b^	15.0	13.0
LOQ (ng/mL)^c^	20.0	18.0

^a^Percentage relative standard deviation

^b^Method detection limit

^c^Limit of quantification

#### Accuracy

The accuracy of the proposed method was assessed by comparing the measured percent recovery of known added amounts of each drug into a blank matrix with those measured by the comparison method [41]. Statistical analysis of the results using Student’s t test and variance ratio *F* test [40] revealed no significant difference in the recoveries of the developed and comparison methods with regard to accuracy and precision, respectively (Tables 3, 4).

**Table 3:** Precision data for the determination of the studied analytes by the proposed method

Parameter	LOR			DSL		
	20.0 ng/mL	100.0 ng/mL	200.0 ng/mL	20.0 ng/mL	100.0 ng/mL	200.0 ng/mL
Intraday precision						
% found	99.85	99.45	98.47	99.7	100.21	99.4
	96.23	100.1	100.078	97.179	99.77	97.82
	97.78	98.19	99.7	98.78	98.87	101.31
Mean	97.95	99.25	99.42	98.55	99.62	99.51
SD	1.82	0.97	0.84	1.28	0.68	1.75
% RSD	1.85	0.98	0.85	1.30	0.69	1.76
% error	1.07	0.57	0.49	0.75	0.40	1.01
Interday precision						
% found	97.25	100.43	99.46	100.58	99.47	98.63
	100.48	97.6	97.84	101.01	97.58	97.01
	99.95	99.85	98.724	99.85	100.94	100.78
Mean	99.23	999.29	98.67	100.48	99.33	98.81
SD	1.73	1.50	0.81	0.59	1.68	1.89
% RSD	1.75	1.51	0.82	0.58	1.70	1.91
% error	1.01	0.87	0.48	0.34	0.98	1.11

Each result is the average of three separate determinations

**Table 4:** Assay results for the determination of the studied analytes in pure form by the proposed and official method [43]

Analyte	Proposed method			Official method [43]		
	Amount taken (ng/mL)	Amount found (ng/mL)	% recovery^a^	Amount taken (µg/mL)	Amount found (µg/mL)	% recovery^a^
LOR	20.0	20.400	102.01	5.0	4.900	98.01
	50.0	50.300	100.61	30.0	30.030	100.1
	80.0	79.700	99.63	50.0	49.990	99.98
	100.0	99.200	99.21			
	200.0	200.400	100.21			
Mean %			100.33			99.37
±SD			1.08			1.18
*t* test^b^	1.196 (2.447)					
*F* test^b^	1.182 (6.944)					
DSL	20.0	20.439	102.20	5.0	4.940	98.71
	50.0	49.684	99.37	30.0	30.260	100.87
	80.0	79.456	98.32	50.0	49.720	99.44
	100.0	100.400	100.35			
	200.0	200.100	100.05			
Mean %			100.26			99.67
±SD			1.17			1.10
*t* test^b^	0.697 (2.447)					
*F* test^b^	1.137 (19.247)					

^a^Each result is the average of three separate determinations

^b^The values between parentheses are the tabulated *t* and *F* values at *P* = 0.05

#### Precision

Intraday and interday precisions were evaluated for each analyte using three different concentrations and three replicates of each concentration. As shown in Table 3, the relative standard deviations were found to be very small which confirms the repeatability and intermediate precision of the developed method.

#### Selectivity

The method selectivity was tested by observing any interference encountered from common Tablet excipients. No interference was observed from any excipient, which indicates high selectivity of the proposed method. Additionally, no interference was encountered from blank human urine, plasma and breast milk matrices without any pretreatment steps.

#### Limit of quantification (LOQ) and method detection limit (MDL)

LOQ and MDL were determined according to ICH Q2 (R1) recommendations [39]. MDL was determined by establishing the minimum level at which the analyte can reliably be detected (signal-to-noise ratio is 3:1) while LOQ was determined by establishing the lowest concentration of analyte that can be determined with acceptable precision and accuracy (signal-to-noise ratio is 10:1). The MDL values were found to be 15.0 and 13.0 ng/mL and the LOQ values were 20.0 and 18.0 ng/mL for LOR and DSL, respectively (Table 2).

#### Robustness

The robustness of the method was evaluated by testing its ability to remain unaffected by small but deliberate variations in the experimental parameters such as variation of: pH of the mobile phase (3.5 ± 0.1), *n*-Butanol concentration (10 ± 0.5%v/v) and SDS concentration (0.15 ± 0.01 M). These deliberate variations did not cause significant change of the peak area of both analytes indicating robustness of the developed method.

### Applications

#### Application to pharmaceutical preparations

**Fig. 3:**
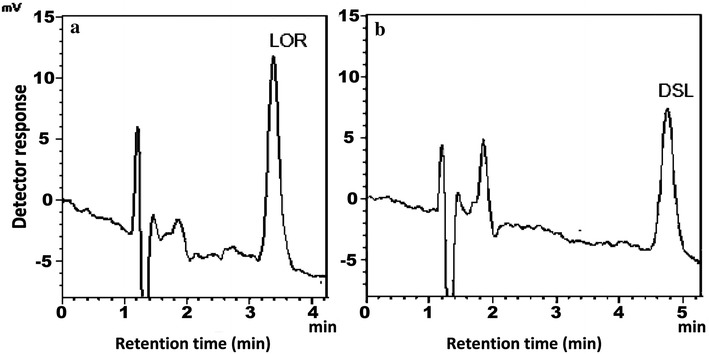
Chromatograms obtained from the application of the proposed method to the analysis of: **a** Loratadine 10 mg Tablets, **b** Desa 5 mg Tablets (analyte concentration: 25 ng/mL for both)

The developed method was successfully applied to the assay of LOR and DSL in their Tablets (Fig. 3). The results obtained are summarized in Tables 4 and 5 showing good agreement with those obtained by the comparison chromatographic method [41]. Statistical analysis of the results obtained using Student’s t test and variance ratio *F* test [39] indicated no significant difference between both them with regard to accuracy and precision, respectively.

**Table 5:** Assay results for the determination of the studied analytes in their different dosage forms by the proposed and official method [41]

Dosage form	Proposed method			Official method [41]		
	Amount taken (ng/mL)	Amount found (ng/mL)	% recovery^a^	Amount taken (µg/mL)	Amount found (µg/mL)	% recovery^a^
Loratadine 10 mg Tablet	50.0	51.636	103.27	5.0	4.950	98.99
	100.0	97.556	97.56	30.0	30.150	100.5
	200.0	200.800	100.41	50.0	49.610	99.22
Mean %			100.41			99.57
±SD			2.86			0.81
*t* test^b^	0.492 (2.776)					
*F* test^b^	12.315 (19)					
Desa 5 mg Tablet	50.0	50.391	100.78	5.0	4.980	99.67
	100.0	99.366	99.37	30.0	30.360	101.2
	200.0	200.100	100.07	50.0	49.390	98.78
Mean %			100.07			99.88
±SD			0.71			1.22
*t* test^b^	0.233 (2.776)					
*F* test^b^	3.014 (19)					

^a^Each result is the average of three separate determinations

^b^The values between parentheses are the tabulated *t* and *F* values at *P* = 0.05

#### Application to biological fluids

LOR undergoes rapid first-pass hepatic metabolism and its major metabolite is DSL. For LOR, the plasma C_max_ is 30.5 ng/mL at 1.0 h after oral administration of 40-mg LOR capsule and for DSL, the plasma C_max_ is 18.6 ng/mL at 2.2 h. About 40% is excreted as conjugated metabolites into the urine, and a similar amount is excreted into the feces. Traces of unmetabolized LOR can be found in the urine [42–44].

After a single oral dose of 40 mg of LOR, average peak milk level (20.4–39.0 ng/mL) occurred at 2.0 h after the dose while the average peak milk level of DSL is in the range of (9.0–29.6 ng/mL) occurred at 5.3 h after the dose [44].

**Fig. 4:**
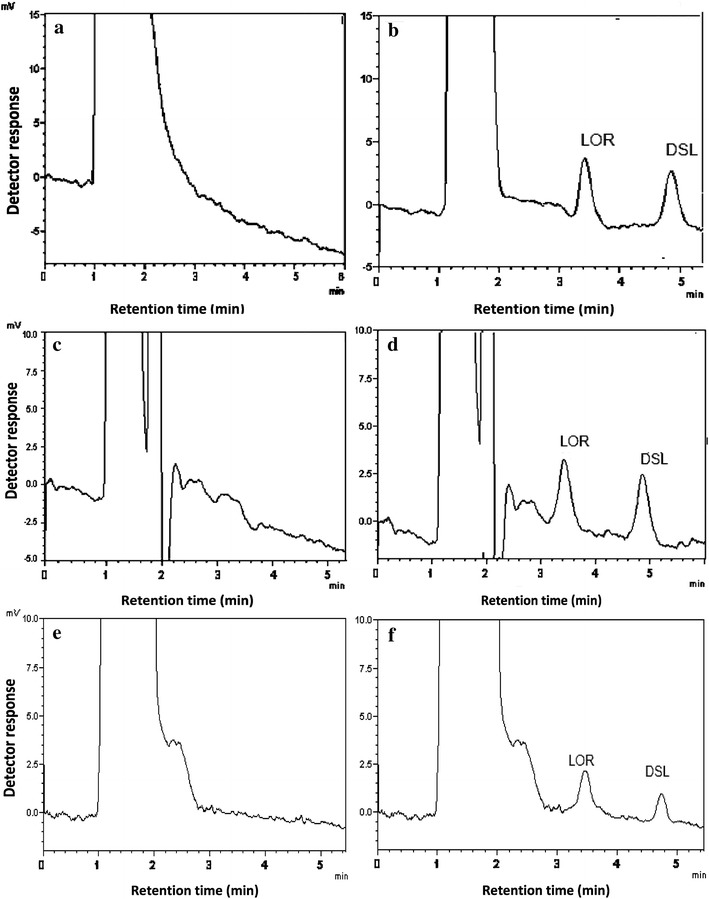
Application of the proposed method to the determination of LOR and DSL in: spiked human urine: **a** Blank urine, **b** spiked urine (analyte concentration: 10 ng/mL), spiked human plasma. **c** Blank plasma, **d** spiked plasma (analyte concentration: 10 ng/mL for both), spiked breast milk. **e** Blank breast milk, **f** spiked breast milk (analyte concentration: 5 ng/mL for both)

Both drugs could be determined in spiked human urine, plasma and breast milk as shown in (Fig. 4). The results are summarized in Table 6. Under the previously described chromatographic conditions, new calibration graphs were established for each drug. The following linear regression equations relating the peak areas to the concentration (ng/mL) were derived:

**Table 6:** Assay results for the determination of the studied analytes in spiked human urine, human plasma and breast milk using the proposed method

Matrix	Parameters	LOR			DSL		
		Amount taken (ng/mL)	Amount found (ng/mL)	Relative recovery (%)^a^	Amount taken (ng/mL)	Amount found (ng/mL)	Relative recovery (%)^a^
Urine	Data	5.0	4.744	94.88	5.0	4.657	93.14
		20.0	20.370	101.85	20.0	20.520	102.60
		50.0	49.800	99.97	50.0	49.800	99.68
	Mean			98.81			98.47
	SD			3.57			4.84
	% RSD			3.61			4.92
	% error			2.08			2.84
Plasma	Data	5.0	4.687	93.74	5.0	4.584	91.68
		20.0	20.477	102.39	20.0	20.631	103.16
		50.0	49.900	99.71	50.0	49.800	99.61
	Mean			98.61			98.15
	SD			4.43			5.88
	% RSD			4.49			5.99
	% error			2.59			3.46
Breast milk	Data	5.0	4.700	94.0	5.0	4.624	92.48
		20.0	20.457	102.29	20.0	20.566	102.83
		50.0	49.900	99.72	50.0	49.800	99.63
	Mean			98.67			98.31
	SD			4.24			5.3
	% RSD			4.3			5.39
	% error			2.48			3.11

^a^Each result is the average of three separate determinations

P = 5.662 + 0.535C (*r* = 0.9999) for LOR in urine, P = 2.888 + 1.527C (*r* = 0.9998) for DSL in urine

P = 8.093 + 0.909C (*r* = 0.9998) for LOR in plasma, P = 4.496 + 1.353C (*r* = 0.9997) for DSL in plasma

P = 8.364 + 0.889C (*r* = 0.9998) for LOR in milk, P = 6.995 + 1.104C (*r* = 0.9998) for DSL in milk

where P is the peak area, C is the concentration of the drug in ng/mL and *r* is the correlation coefficient.

## Conclusions

The current study represents a novel MLC method using a monolithic column for the simultaneous determination of LOR and DSL which is the major metabolite of LOR as well as one of its impurities. The developed method is able to separate both drugs with high resolution factor and high efficiency within a very short analysis time (less than 5 min). The method can be successfully applied for the assay of both analytes in their pharmaceutical preparations and in spiked human urine, plasma and breast milk without prior extraction procedures. The validation criteria of the developed MLC method indicate its reliability and allow its application in quality control analyses. Moreover, it can be utilized as a simple time-saving alternative to the official pharmacopeial method for testing DSL as a potential impurity in LOR bulk powder.

## Abbreviations

LOR: loratadine
DSL: desloratadine
SDS: sodium dodecyl sulfate
MLC: micellar liquid chromatography
TEA: triethylamine
ICH: international conference on harmonization
LOQ: limit of quantification
MDL: method detection limit
